# Reduced penetrance in hereditary movement disorders

**DOI:** 10.1515/medgen-2022-2132

**Published:** 2022-08-12

**Authors:** Christine Klein, Frank Kaiser

**Affiliations:** Institute of Neurogenetics, University of Lübeck, 23538 Lübeck, Germany; Institute of Human Genetics, University of Duisburg-Essen, University Hospital Essen, 45122 Essen, Germany

Reduced penetrance is a well-documented conundrum in hereditary diseases, where it initially emerged from case and family studies of monogenic disorders. Overall, penetrance of disease-causing alleles and genotypes is likely much lower than previously thought. One possible explanation of this discrepancy is that classic studies of inherited diseases were based on the presence of the pathological phenotype in multiple family members, i. e., full or at least high penetrance of the disease, thereby ‘avoiding’ the penetrance issue or at least skewing penetrance estimates towards higher percentages. At an unprecedented scale, NGS efforts allow to assess penetrance of putatively pathogenic mutations and gene variants from the reverse perspective – based on large numbers of presumably non-diseased individuals – and to discover protective alleles.

Formally, ‘penetrance’ is defined as the conditional probability of being affected with disease X given a specific genotype. Penetrance is closely related and sometimes even confused with ‘variable expressivity’: While penetrance relates to the proportion of a population actually expressing the phenotype at all if a given genetic variant is present, expressivity describes the extent to which the phenotype is then expressed. Admittedly, in clinical practice and research, it is often difficult – if not impossible – to firmly distinguish between reduced penetrance and variable expressivity: Do subtle signs not (yet) allowing to establish the diagnosis of a given disease ‘count’ as a manifesting phenotype? How about preclinical or paraclinical disease manifestations not detectable with the unaided eye? Furthermore, penetrance is often age-dependent. For example, while pathogenic variants causative of Parkinson’s disease (PD) often have reduced penetrance, not all at-risk individuals live up to the age of disease manifestation which may be only late in life.

In view of the surprisingly large number of individuals carrying allegedly pathogenic variants, but who eventually do not develop the disease in question in their lifetime, the importance of reduced penetrance appears to have been substantially underestimated and represents an important challenge in medical genetics and personalized medicine. More generally speaking, there is a preoccupation with disease susceptibility, whereas the concepts of protection against disease, delay of its onset age, or attenuation of its severity have thus far been largely neglected within the genomic research community. Also, the advent of gene-targeted therapies for selected hereditary movement disorders has made the identification of penetrance-modifying factors all the more critical and timely.

In the present volume, hereditary movement disorders, such as monogenic PD and dystonia serve as a use case to i) explore the role of penetrance (and variable expressivity), ii) highlight examples of mechanistic insights explaining reduced penetrance, and iii) discuss models and research strategies to improve our understanding of reduced penetrance and its translational role.

In their article ‘Factors influencing reduced penetrance and variable expressivity in X-linked dystonia-parkinsonism’ Laabs et al. discuss a powerful example of a monogenic movement model disease where strong genetic modifiers influencing age-at-onset-related penetrance and disease severity have been identified. LRRK2 mutations are the most common known cause of PD and penetrance is markedly reduced. Gruenewald et al. describe the known molecular mechanisms defining penetrance of LRRK2-associated PD. Cellular models as well as animal models for the study of reduced penetrance are reviewed by Seibler et al. and Vos and Morais, respectively. Reflecting the importance of a multimodal approach to study reduced penetrance, Diaw et al. address the emerging role of a systems biology approach using THAP1-linked dystonia as an example. Finally, Kappen and colleagues provide a systematic review of Mendelian randomization studies on PD and, more generally speaking, highlight the utility of Mendelian randomization for the study of reduced penetrance.

